# Socioeconomic and sociodemographic differences in the consequences of the COVID-19 pandemic and their impact on self-rated health and mental well-being: results from a cross-sectional study in Germany

**DOI:** 10.1186/s12889-025-23698-w

**Published:** 2025-07-22

**Authors:** Birgit Babitsch, Cristina Ciupitu-Plath

**Affiliations:** 1 Department of New Public Health, Institute of Health Research and Education, School of Human Sciences, Osnabrück University, Osnabrück, Germany; 2 Department of Public Health, Bastyr University, Kenmore, WA USA

**Keywords:** Socioeconomic status, Sociodemographic factors, Health inequity, COVID-19, Self-efficacy, Resources, Burden, Self-rated health, Mental well-being

## Abstract

**Background:**

Although the COVID-19 pandemic has demonstrably led to an increase in health inequities, only a few studies have analyzed their underlying mechanisms by taking into account socioeconomic status and sociodemographic differences at the same time. Similarly, only few studies have explored the impact of COVID-19 containment measures on inequities in living conditions, health-related risks, and coping resources. This study aims to address these gaps by exploring the complex associations of socioeconomic and sociodemographic factors with changes in life circumstances, pandemic-related experiences, self-rated health, and well-being among adults living in Germany.

**Methods:**

A total of 2,123 adults (women: 49.8%, men: 50.2%) living in Germany participated in the cross-sectional online study ExCo:Well between July and August 2022. The survey included questions on socioeconomic status, sociodemographic factors, social circumstances, resources and burdens, as well as health outcomes. The data were analyzed using bivariate and multivariable logistic regression analyses.

**Results:**

Our results show significant disparities in self-rated health and mental well-being based on socioeconomic status. For sociodemographic differences, the results are mixed, with only women consistently showing worse health outcomes than men. Immigration status played a limited role. Although measures to contain the COVID-19 pandemic more commonly affected the life and work conditions of more privileged participants, socioeconomically disadvantaged participants experienced higher burdens and had fewer coping resources. Logistic regression analyses showed that health inequities decreased when resources and burdens were considered.

**Conclusions:**

By covering the whole period of the COVID-19 pandemic, our data allow for an overall assessment of this critical time as well as a better understanding of mechanisms underlying health inequities. Our findings suggest that more important than the number of government-induced social changes is their quality and their potential to negatively impact material and social livelihoods in the long run. To improve health equity, tailored social security and health promotion interventions need to be systematically integrated in pandemic or crisis response plans.

**Supplementary Information:**

The online version contains supplementary material available at 10.1186/s12889-025-23698-w.

## Introduction

SARS-CoV-2 affected all societies and tested their resilience in dealing with this unknown virus that caused over 7 million deaths worldwide [1], and more than 174,000 fatalities in Germany alone [2]. In addition to large-scale vaccination programs, broader public health and social measures (PHSM) were implemented to contain the COVID-19 pandemic, including curfews, physical distancing, business closures, and mask wearing. In Germany, these strategies were deployed alongside numerous economic policy measures designed to alleviate the financial burden of the pandemic.

### Impact of the COVID-19 pandemic on self-rated health and well-being

Apart from its high death toll, the COVID-19 pandemic also contributed to significant reductions in self-rated health [3–5] and well-being [6–13]. Although the magnitude of these associations varied across different samples [8, 10–12, 14–17], taken together they deliver a well-rounded overview of the health impacts of the pandemic, as self-rated health and well-being are two of the most commonly used health indicators [18, 19]. Despite their partial overlap and largely subjective nature, these two constructs provide distinct information about individual health. Albeit usually relying on a single-item assessment, self-rated health is a broad and largely stable construct reflecting individuals’ perception of their functioning at the intersection of physical, mental, and social health [20]. In turn, well-being refers to a state of positive mental health, in which individuals can reach their full potential and effectively contribute to their community as socially and psychologically well-adjusted and functioning members [21].

### Sociodemographic differences in the impact of the COVID-19 pandemic on self-rated health and well-being

The effects of the COVID-19 pandemic on self-rated health and well-being have varied not only in magnitude, but also across different population groups [11, 13, 22]. Typically, women [5, 23], older age groups [5] and people with low educational attainment [4, 5], lower income or financial insecurity [4, 24, 25] were less likely to rate their health as good during the COVID-19 pandemic. Similarly, most studies found an association between poorer well-being and female gender [6, 8, 10, 13, 22, 26–28], younger age [6, 8, 11, 13, 22], lower educational attainment [8, 10, 11, 26, 27, 29], lower household incomes [13], living in socioeconomically disadvantaged neighborhoods [6, 7] and having a chronic disease [13, 26, 27].

### Explanatory factors in self-rated health and well-being in the COVID-19 pandemic

Overall, both the direct threat posed by SARS-CoV-2 and PHSM consequences such as financial insecurity and reduced social contacts contributed to reductions in self-rated health [3, 17, 24] and mental well-being [7, 22, 30]. Although PHSM typically had a greater impact on socioeconomically disadvantaged populations [31, 32], some studies found that increased risks were only associated with some PHSM (e.g., job loss, worsening financial situation) [33, 34], but not others (e.g., reductions in work hours) [33].

In contrast, coping strategies such as exercising or spending time with family were positively associated with well-being [26] and individuals with a high functional coping profile also had the highest well-being scores [35]. People reporting lower well-being used significantly more distraction strategies such as home entertainment, while those with higher well-being dedicated more time to self-care, family and friends [36]. Overall, the ability to cope with restrictions in social life was lower in socioeconomically disadvantaged populations [34].

### Health inequities before and during the COVID-19 pandemic

Numerous studies have consistently shown that socioeconomic and sociodemographic differences play an important role in shaping individuals’ chances of living in good health [37–40]. In Germany, even prior to the pandemic, societal factors such as the increase in income inequality between 1994 and 2014 were associated with a significant decline in self-rated health [41] and life expectancy [42]. At the same time, individual characteristics like gender [43, 44] and migration [45, 46] are strong predictors of health status among people living in Germany.

The conceptual framework proposed by the WHO Commission on Social Determinants of Health (CSDH) clearly distinguishes between such structural (e.g., policies, socioeconomic and sociodemographic factors) and intermediary (e.g., living conditions, psychosocial factors) determinants of health [47]. Drawing on this framework, it is important to note that the COVID-19 pandemic occurred within the context of preexisting social and health inequities associated with greater vulnerability and higher risk of poorer health outcomes among socioeconomically disadvantaged groups, a phenomenon described as a syndemic pandemic [48–54]. A higher risk of contracting COVID-19, a more severe course of disease, and higher mortality [2, 31, 48, 49, 55–66] have been found in socioeconomically disadvantaged groups, a pattern also noted in Germany [67, 68].

### Factors driving health inequity during COVID-19

The factors underlying observed COVID-19 disparities are clearly rooted in much of the same mechanisms that are responsible for endemic health inequities [48, 49, 54, 64, 69, 70]. As a result of the drastic social changes during the COVID-19 pandemic, structures of social disparity were likely reinforced or exacerbated [54]. Accordingly, a COVID-19-specific adaptation of the CSDH [70] included PHSM as structural determinants and the different risks of exposure to SARS-CoV-2 as intermediary determinants of health.

The higher vulnerability of socioeconomically disadvantaged groups to the COVID-19 pandemic can therefore be understood as a double burden resulting from (1) a higher pandemic impact on the structural and intermediary determinants of health [47] and (2) a poorer health status prior to the pandemic [37–40]. Specifically, socioeconomically disadvantaged groups had a higher probability of experiencing a deterioration in their living conditions, more risks, and fewer resources and coping mechanisms [48, 49, 54, 70]. Against this background, the amount and quality of coping resources available are likely to play a crucial role in the emergence of health inequities [71–73].

Therefore, distinguishing between ex-ante and ex-post social and health inequities was suggested as a helpful mechanism for working out the inequitable structures and individual possibilities that affected the handling of dramatic pandemic-related changes or resulted from these changes [74]. Using these frameworks can help identify specific strategies to adequately support socioeconomically disadvantaged groups during future public health crises and avoid an increase in health inequities in the long term.

### Study aim, research questions, and hypotheses

While a growing body of research has investigated the role of socioeconomic status and sociodemographic factors in shaping experiences during the COVID-19 pandemic, detailed analyses of the interplay between these factors, changes in living conditions, and health-related resources remain limited. Existing studies often focus on single determinants or broad indicators, leaving a gap in understanding the complex inequities that emerged during the pandemic as illustrated by the concept of syndemic pandemic [48–54]. Further research is needed to assess the role of health-related resources and their interplay with risk factors in a differentiated manner, which is crucial to elucidate health inequities associated with the COVID-19 pandemic.

To address this research gap, our study provides a detailed examination of the complex associations between socioeconomic and sociodemographic factors, changes in life circumstances, pandemic-related experiences, and self-rated health and well-being among adults living in Germany. The main research question explored in this study is: How is the relationship of socioeconomic and sociodemographic factors with self-rated health or mental well-being influenced by pandemic-related burdens and resources in Germany?

The following hypotheses were tested:

1. People with low socioeconomic status report poorer self-rated health and lower mental well-being compared to people with high socioeconomic status.
2. Older people, women, as well as immigrants and their (direct) descendants report poorer self-rated health and lower mental well-being compared to younger people, men, and people without an immigration history.
3. The social consequences and burdens associated with the COVID-19 pandemic are greater among people with low socioeconomic status than among those with high socioeconomic status.
4. Resources are lower among people with low socioeconomic status than among those with high socioeconomic status.
5. Resources and burdens influence the association of socioeconomic and sociodemographic factors with self-rated health and mental well-being, respectively.

With its rich data on social determinants, changes in living conditions, and specific health-related resources, this paper contributes to a deeper understanding of health inequities in Germany and can help identify opportunities to support population health under challenging circumstances.

### Methods

#### Study design

The data originate from the cross-sectional, online study “Experiencing the COVID-19 Pandemic: Association between Resources, Social Health, and Well-Being (ExCo:Well)”. The impact of the COVID-19 pandemic extended beyond direct health threats to significantly alter daily life due to PHSM. Recognizing this profound and unequal impact ExCo:Well aimed to investigate 1. how PHSM affected individuals’ daily life experiences, 2. whether these experiences were driven by socioeconomic and sociodemographic differences, and 3. how all these factors affected self-rated health and mental well-being. This study builds on and expands existing research by providing a detailed description of changes related to PHSM, COVID-19 related burdens, and COVID-19 specific and general resources (e.g., general self-efficacy).

Beginning in March 2020, numerous measures to contain the COVID-19 pandemic were enacted in Germany by the government at the federal and state level, including curfews, physical distancing, the closure of businesses, and mask wearing. The measures were dynamically adapted to the SARS-CoV-2 infection rates and have been significantly scaled back since May 2022 and completely eliminated as of 8 April 2023 [75, 76]. ExCo:Well was conducted in Germany from 22 July 2022 to 22 August 2022, generating insights into participants’ views and experiences of the entire COVID-19 pandemic and the first months after pandemic restrictions were loosened. Data were collected by an external survey company (respondi, https://mingle.respondi.com) from members of an online panel that has been successfully used in other population-based studies during the COVID-pandemic [77].

The survey included 49 questions, divided into the following sections: COVID-19 pandemic experiences, COVID-19 related personal and societal changes, COVID-19 related (digital) health literacy, COVID-19 specific and general resources, self-rated health, mental well-being, personal attitudes, COVID-19 specific burdens, socioeconomic, and sociodemographic factors. Wherever possible, validated instruments were used in the development of the survey; the development of new questions specifically to assess COVID-19-related aspects followed a structured qualitative approach including cognitive interviews (see Operationalization section for details). The study is reported according to the STROBE statement [78].

### Study sample

All participants were members of an online panel who were informed about the study and invited to participate by an external survey company. A total of 4,753 people accepted the invitation and met the inclusion criteria, of which 3,175 completed the online questionnaire in full.

Quota sampling was used to achieve representativeness in terms of age (18 to 74 years) and gender (crossed), as well as federal state based on Eurostat 2020 data. Moreover, a quota of 30% was set for people with low educational attainment to ensure sufficient data were available for stratified analyses. This corresponds to the distribution of educational attainment among people living in Germany [79].

A sample size was calculated for regression analysis, requiring a minimum of 1,169 participants (power of 0.9, effect size of 0.02, significance level of α = 0.05) [80]. However, to allow for stratified analyses (e.g., by immigration status or educational attainment), it was decided to increase the sample size. The final sample size of 2,123 was randomly selected by the external survey company, which is appropriate in terms of quotas (see above) and data quality.

After completion of the data collection, an anonymized data set was provided to the research team by the external survey company. All the data protection requirements were guaranteed by the external company. The study was approved by the ethics committee of Osnabrück University (Ethik-41/2022, 28.6.2022).

### Operationalization

#### Sociodemographic and socioeconomic variables

In this study, socioeconomic (education, income, subjective social status) and sociodemographic factors (age, gender, immigration status) were assessed using various measures. Age was divided into four groups in line with federal health reporting procedures [81]: 18–29, 30–44, 45–64, and 65–74 years. Gender was self-reported by participants as ‘male’ or ‘female’. Immigration status was assessed according to the recommended standards in Germany [82–84]. A distinction was made between two categories: (a) immigrants and their (direct) descendants (i.e., participants not born in Germany or participants born in Germany with both parents born outside of Germany) and (b) people without an immigration history (i.e., participants and one or both parents born in Germany) [84].

Education was assessed using the CASMIN classification [85] and divided into basic, medium and higher educational attainment [86]. Subjective social status (SSS) was measured using the MacArthur Scale of Subjective Social Status [87] as a self-assessment on a 10-step ladder. Participants’ responses were then categorized as low (scale values 1–4), medium (scale values 5–6) and high SSS (scale values 7–10) [88]. Income was assessed as the monthly net household income in line with the demographic reporting standard [89]. In addition, participants were given the option to offer ‘no response’. For the analysis, income categories were combined into four monthly net household income groups (< €1.000, €1.000-2.999; €3.000-4.999; >=€5.000). An adjustment of income level by household size was not possible. Information on partnership and parental status, as well as community size was also requested.

#### Living situation during the COVID-19 pandemic

Several aspects of participants’ living situation were surveyed to assess the changes they have experienced due to the COVID-19 pandemic in Germany. Participants were asked (a) to provide an overall assessment of how easy it was for them to navigate the COVID-19 pandemic and (b) to indicate how much their life circumstances changed during this time. Both aspects were assessed using 5-point Likert scales with the following answer options (a) 1= ‘very easy’, 2= ‘easy’, 3= ‘neither easy nor difficult’, 4= ‘difficult’, 5= ‘very difficult’, (b) 1= ‘not at all’, 2= ‘rather weak’, 3= ‘neither strong nor weak’, 4= ‘rather strong’, 5= ‘very strong’), which were combined into three groups for analysis (1= ‘rather difficult or rather strong’; 2= ‘neither’; 3= ‘rather easy or rather weak’).

In addition, the prevalence of the most common PHSM in Germany (e.g., working from home, extra work, closure of companies or loss of employment, work hour reduction, reduction in overtime and/or holiday, delegation of other tasks, and homeschooling) [75, 76] were assessed (response categories: ‘yes’, ‘no’, ‘not applicable’). Each category was analyzed separately. A limitation of this assessment is that it does not provide information on the magnitude and duration of these changes.

In order to gauge how participants experienced the entire COVID-19 pandemic, they were asked to indicate the extent to which they felt threatened, burdened, and restricted during this time. For this purpose, they were given five response options ranging from ‘very strong’ to ‘not at all’. This 5-point Likert scale was reduced to three values for analysis. Moreover, to explore how the participants’ living situation recovered after the PHSM were scaled back, they were asked to estimate how similar their current living situation was compared to their living situation before the COVID-19 pandemic. The extent to which participants’ life situation had returned to normal was assessed in two ways: first, using a 5-point Likert scale (response categories: ‘not at all’ to ‘very strong’), and second, using a slider scale that allowed respondents to select any number between 0 and 100%.

#### Burdens during the COVID-19 pandemic

The COVID-19 Pandemic-related Burden Scale (CBS) was created to assess specific burdens on social life resulting from PHSM to contain the COVID-19 pandemic in Germany. The scale items were developed in 2021 based on a comprehensive literature review, including both scientific and popular sources, and validated through cognitive interviews (*N* = 5). The items capture not only changes in material living conditions, but also burdens experienced in daily life. The final CBS included 12 items rated on a 5-point Likert Scale (1= ‘completely disagree’, 2= ‘mostly disagree’, 3= ‘somewhat agree’, 4= ‘mostly agree’, 5= ‘completely agree’). In addition, the response category ‘does not apply’ was offered. Because of their similar meaning, the response categories ‘does not apply’ and ‘strongly disagree’ were merged in the analysis. To estimate the overall burden, the CBS score was calculated by adding all 12 items (range 12–60). Reliability was good, with Cronbach’s alpha = 0.869.

Principal component analysis (PCA) revealed two independent factors of the CBS: (1) material burden (range: 6–30, Cronbach’s alpha = 0.833) and (2) psychosocial burden (range: 6–30, Cronbach’s alpha = 0.851) (see Additional file 1, Supplementary Table 1).

#### Resources during the COVID-19 pandemic

The COVID-19 Pandemic-Related Resources and Coping Scale (CRCS) was developed in 2020 based on a comprehensive literature review of coping strategies during the pandemic, including both scientific and popular sources, and validated through cognitive interviews (*N* = 5). The final CRCS included 12 items rated on a 5-point Likert scale (1= ‘does not apply at all’, 2= ‘applies to a low extent’, 3= ‘applies to a moderate extent’, 4= ‘applies to a large extent’, 5= ‘applies to a very large extent’).

To estimate the overall level of coping, the CRCS score was calculated by adding all 12 items (range 12–60). Reliability was good, with Cronbach’s alpha for standardized items = 0.881. For the CRCS, a PCA was performed, identifying two factors: (1) self-focused coping strategies (range: 7–35, Cronbach’s alpha = 0.856) and (2) social engagement (range: 5–25, Cronbach’s alpha = 0.756) (see Additional file 1, Supplementary Table 2).

Participants’ general self-efficacy (GSE) was assessed using the 10-item scale developed by Jerusalem & Schwarzer [90]. The items measure optimistic expectations of competence, i.e., the confidence that one can master a difficult situation, whereby success is attributed to one’s own abilities. A total score was generated (range 10 to 40), with higher values indicating higher self-efficacy. A dichotomous variable (low vs. high self-efficacy) was created using the value 29 as a cut-off point [90]. Reliability was excellent (Cronbach’s alpha = 0.926).

#### Self-rated health and mental well-being

To describe the health status of the sample, participants’ self-rated health [91] and the presence of a chronic disease (‘yes’/’no’) were assessed. Using a 5-point Likert scale, the participants were asked to rate their overall health status from ‘very good’ to ‘very poor’ [91]. A dichotomous variable was created to distinguish between ‘rather good’ (response categories: ‘good’ and ‘very good’) and ‘rather poor’ (response categories: ‘satisfactory’, ‘poor’ and ‘very poor’) self-rated health.

The WHO-5 questionnaire [92] was used to survey mental well-being. All five items were added to obtain a total score (range 0–25). Following Brähler et al. [93], a dichotomous variable was created with a cut-off point at 13. Reliability was excellent (Cronbach’s alpha = 0.923).

### Data analysis

The data were analyzed using IBM SPSS Version 29.0. Univariate and bivariate analyses were carried out for the descriptive analysis. The median was used when the data were not normally distributed, as determined by the Kolmogorov-Smirnov test.

All analyses were stratified by socioeconomic (educational attainment (CASMIN), monthly net household income, and SSS) and sociodemographic (age, gender, and immigration status) variables. Significance was tested using the Pearson chi^2^-test, the Mann-Whitney-U test, and the Kruskal-Wallis test, as appropriate.

Factor analyses were conducted to examine the properties of the new scales (CBS, CRCS). The reliability of all scales was tested with Cronbach’s alpha.

Multivariable logistic regression models were calculated for self-rated health (1 = rather good) and for mental well-being (1 = high) as dependent variables. The independent variables were selected based on theoretical considerations and included block-wise: (1) socioeconomic and sociodemographic variables, (2) resources and burdens, and (3) presence of a chronic disease.

The significance level was set at 0.05 for all analyses.

## Results

### Study sample

Female and male participants were almost equally represented in the sample, reflecting the gender distribution of the population living in Germany. On average, participants were 46.3 years old; the largest age group was between 45 and 65 years old (40.0%). In total, 9.4% of the participants were immigrants or their (direct) descendants. A basic level of education was attained by 31.3%, a medium level by 47.7% and a high level by 21.0% of the participants. Two-thirds of the participants had a monthly net household income between 1,000 and 4,999 euros; 7.6% did not answer this question. SSS was classified as low by 29.8% of participants and as high by 32.5%. A total of 61.4% lived in a partnership, 55.2% had children and 42.4% lived in a place with fewer than 20,000 inhabitants. A SARS-CoV-2 infection was reported by 37.4% of the participants (see Table 1).

**Table 1:** Description of the study sample

Characteristics (*N* = 2,123)	*N*	%
**Gender**		
Female	1,058	49.8
Male	1,065	50.2
**Age groups**		
18–29 years	406	19.1
30–44 years	567	26.7
45–64 years	849	40.0
65–74 years	301	14.2
**Age** (in years, median)	2,123	47.0
**Age** (in years, mean, (std. dev))	2,123	46.3 (15.4)
**Immigrants and their (direct) descendants**	199	9.4
**Educational attainment (CASMIN)**		
Basic	665	31.3
Medium	1,013	47.7
Higher	445	21.0
**Monthly net household income**		
No answer	161	7.6
Up to under 1,000 euros	243	11.4
1,000 to 2,999 euros	947	44.6
3,000 to 4,999 euros	576	27.1
5,000 euros and more	196	9.2
**SSS**		
Low	633	29.8
Medium	800	37.7
High	690	32.5
**Living in partnership**	1,303	61.4
**Children**	1,171	55.2
**Community size**		
Fewer than 20,000 inhabitants	900	42.4
**Infection with SARS-CoV-2** (self-reported)	793	37.4

### Changes in and perceptions of personal living conditions during the COVID-19 pandemic

The PHSM to contain the COVID-19 pandemic have drastically changed living conditions and revealed numerous patterns of inequity. Considering the whole period of the COVID-19 pandemic, half of the participants (52.5%) reported experiencing rather significant life changes. This was significantly more common among younger participants (60.8%) and those with a low SSS (56.6%), but also among those with higher education (55.5%).

Overall, 30.3% of respondents worked from home, 19.2% reported additional work, 11.3% lost their jobs, 15.1% experienced work hour reductions, 14.9% had to reduce overtime or take leave, 15.4% were given new responsibilities, and 18.8% had to take on homeschooling or childcare responsibilities. Many of these life changes were more commonly reported by participants with higher socioeconomic status.

The COVID-19 pandemic was perceived as threatening by 40.3% of participants, burdening by 49.8%, and restrictive by 51.4%. These perceptions varied more across sociodemographic groups, but little across socioeconomic strata, where only significant differences with regard to the perceived burden of the COVID-19 pandemic were noted. Specifically, a higher percentage of older participants perceived the pandemic as a threat, yet significantly more participants in younger age groups felt burdened by the pandemic. Female participants fared worse than male participants on all assessed measures.

Navigating the COVID-19 pandemic was retrospectively rated as being rather difficult by a third of the participants. Younger age groups, female participants, and participants with lower SSS were significantly more likely to report this.

At the time of data collection, 43.3% of the study participants reported that their current living situation had started to become more similar to their living situation before the COVID-19 pandemic. This is consistent with participants’ overall assessment that their current living situation corresponds to 79% (median) of their living situation before the COVID-19 pandemic. Recovery rates were significantly lower among participants who were younger, female, and had lower SSS. For example, 50.9% of participants with high SSS vs. 34.8% of participants with low SSS reported that they had returned to their usual, pre-pandemic living situation (see Table 2, see Additional file 2, Supplementary Table 2_1 for the results stratified for immigration status, educational attainment (CASMIN) and monthly net household income).

**Table 2:** Experiences and changes in the life situation through the COVID-19 pandemic stratified by age, gender and SSS

Characteristics (*N* = 2,123)	*N*	Age					Gender			SSS				Total
		18–29	30–44	45–64	65–74	*p* ^1^	M	F	*p* ^1^	L	M	H	*p* ^1^	
**Changes in life through measures to contain the COVID-19 pandemic**						< 0.001			0.038				0.016	
Rather strong^a^ (%)	1,115	60.8	57.8	48.3	43.2		50.6	54.4		56.6	52.9	48.4		52.5
**Working from home** (%)						< 0.001			< 0.001				< 0.001	
Yes	644	56.7	40.4	20.7	3.0		34.8	25.8		16.6	28.2	45.4		30.3
No	606	24.1	32.5	32.0	17.3		29.1	28.0		25.9	31.9	27.1		28.5
Not applicable	873	19.2	27.2	47.2	79.7		36.1	46.2		57.5	39.9	27.5		41.1
**Extra work** (%)						< 0.001			< 0.001				< 0.001	
Yes	408	26.1	30.3	14.7	1.7		19.2	19.3		14.1	21.0	21.9		19.2
No	945	51.2	49.6	46.3	20.9		49.3	39.7		36.2	45.6	50.9		44.5
Not applicable	770	22.7	20.1	39.0	77.4		31.5	41.0		49.8	33.4	27.2		36.3
**Loss of employment** (%)						< 0.001			< 0.001				< 0.001	
Yes	240	15.0	13.9	10.4	4.0		12.5	10.1		12.3	11.4	10.3		11.3
No	1,116	58.9	64.2	52.4	22.6		57.4	47.7		39.7	54.9	61.7		52.6
Not applicable	767	26.1	21.9	37.2	73.4		30.1	42.2		48.0	33.8	28.0		36.1
**Work hour reductions** (%)						< 0.001			< 0.001				< 0.001	
Yes	321	19.2	21.2	13.7	2.3		16.9	13.3		13.3	15.0	17.0		15.1
No	1,016	53.7	57.7	47.8	21.6		52.0	43.7		37.3	49.9	55.2		47.9
Not applicable	786	27.1	21.2	38.5	76.1		31.1	43.0		49.4	35.1	27.8		37.0
**Reduction overtime and/or holiday** (%)						< 0.001			< 0.001				< 0.001	
Yes	317	19.0	22.0	13.1	1.3		16.8	13.0		11.2	13.8	19.7		14.9
No	1,023	55.2	57.3	48.3	21.3		51.2	45.2		38.5	52.3	52.3		48.2
Not applicable	783	25.9	20.6	38.6	77.4		32.0	41.8		50.2	34.0	28.0		36.9
**Delegation of other tasks** (%)						< 0.001			< 0.001				< 0.001	
Yes	328	26.8	20.8	11.4	1.3		15.9	15.0		12.0	15.3	18.8		15.4
No	1,020	48.3	57.5	50.4	23.3		52.0	44.0		38.2	50.2	54.5		48.0
Not applicable	775	24.9	21.7	38.2	75.4		32.1	40.9		49.8	34.5	26.7		36.5
**Homeschooling** (%)						< 0.001			< 0.001				< 0.001	
Yes	400	20.7	35.8	12.1	3.3		18.8	18.9		16.3	18.0	22.2		18.8
No	787	44.6	35.8	40.3	20.3		42.1	32.0		28.1	39.5	42.5		37.1
Not applicable	936	34.7	28.4	47.6	76.4		39.2	49.1		55.6	42.5	35.4		44.1
**Perceived threat from the COVID-19 pandemic**						0.013			0.003				0.390	
Rather strong^a^ (%)	855	40.9	39.3	38.6	45.8		36.8	43.8		42.8	38.5	40.0		40.3
**Perceived burden of the COVID-19 pandemic**						< 0.001			< 0.001				< 0.001	
Rather strong^a^ (%)	1,057	58.6	52.7	46.9	40.5		44.1	55.5		55.9	49.8	44.2		49.8
**Perceived restrictions due to the COVID-19 pandemic**						< 0.001			< 0.001				0.092	
Rather strong^a^ (%)	1,092	59.9	58.7	47.6	37.2		47.1	55.8		55.1	50.0	49.7		51.4
**Overall assessment of dealing with the COVID-19 pandemic**						0.010			< 0.001				< 0.001	
Rather difficult^b^ (%)	759	42.9	37.2	33.6	29.6		31.6	39.9		43.4	34.3	30.4		35.8
**Change in living situation due to the relaxation of COVID-19-related restrictions**						< 0.001			0.456				< 0.001	
Rather strong^a^ (%)	919	54.9	50.4	37.7	29.9		43.3	43.3		34.8	43.5	50.9		43.3
**Percentage of adjustment between current living situation and everyday life before the coronavirus pandemic**						<.001^3^			.020^2^				<.001^3^	
Median	2,123	70.0	75.0	80.0	80.0		80.0	77.0		71.0	77.5	80.0		79.0

^1^ Pearson Chi^2^-Test unless otherwise noted; ^2^ Mann-Whitney-U test; ^3^ Kruskal-Wallis test; ^a^ Includes the response categories: ‘rather strong’ and ‘very strong’; ^b^ Includes the response categories: ‘difficult’ and ‘very difficult’;

Gender: *M *Male, *F *Female, SSS: *L *Low, *M *Medium, *H *High

### Experienced burdens during the COVID-19 pandemic

The majority of participants experienced burdens related to their personal, social and material situation, as measured by the COVID-19 Pandemic-related Burden Scale (CBS). Considering the entire COVID-19 pandemic, 22.5% of the participants felt that their livelihood was threatened and 34.3% assumed that it would take a long time to return to their previous living standard. A high percentage of participants experienced challenges in their social lives and in coping with changed living conditions. Younger age groups, female participants, immigrants and their (direct) descendants, and those with low SSS perceived their life situation as more challenging, with few exceptions. The overall perceived burden was low, with a median CBS score of 24.0 (range 12–60). This was also found for both the Psychosocial burden subscale (median = 15.0, range 6–30) and the Material burden subscale (median = 8.0, range 6–30). Both types of burdens were significantly greater among younger age groups and participants with low SSS (see Table 3, see Additional file 2, Supplementary Table 3_1 for the results stratified for immigration status, educational attainment (CASMIN) and monthly net household income).

**Table 3:** Experienced burdens measured by the COVID-19 Pandemic-related burden scale (CBS) during the COVID-19 pandemic stratified by age, gender and SSS

Characteristics (*N* = 2,123)	*N*	Age					Gender			SSS				Total
		18–29	30–44	45–64	65–74	*p* ^1^	M	F	*p* ^1^	L	M	H	*p* ^1^	
Livelihood threatened^a^ (%)	477	25.9	28.2	22.5	7.0	< 0.001	25.1	19.8	0.004	31.1	19.1	18.4	< 0.001	22.5
Long time to get back to previous standard of living^a^ (%)	728	38.9	37.6	34.2	22.3	< 0.001	34.9	33.6	0.534	47.9	32.1	24.3	< 0.001	34.3
Housing situation worsened^a^ (%)	368	24.9	23.3	14.4	4.3	< 0.001	20.6	14.1	< 0.001	20.9	16.1	15.5	0.019	17.3
Applied for state benefits^a^ (%)	428	26.8	24.2	18.1	9.3	< 0.001	23.6	16.7	< 0.001	27.3	17.4	16.8	< 0.001	20.2
Gave up own business^a^ (%)	238	18.2	17.1	6.6	3.7	< 0.001	15.4	7.0	< 0.001	8.2	11.3	13.9	0.005	11.2
Not reaching full potential at work^a^ (%)	424	29.1	27.0	16.4	4.7	< 0.001	22.8	17.1	0.001	22.4	20.0	17.7	0.097	20.0
Felt socially isolated^a^ (%)	880	54.9	49.6	37.0	20.6	< 0.001	38.2	44.7	0.002	52.1	39.0	34.5	< 0.001	41.5
Exhausted due to the multiple challenges^a^ (%)	913	56.9	54.3	38.3	16.3	< 0.001	39.9	46.1	0.004	48.8	43.3	37.4	< 0.001	43.0
Not part of a social group^a^ (%)	834	50.2	44.3	36.6	22.6	< 0.001	37.4	41.2	0.070	49.8	37.6	31.6	< 0.001	39.3
Not able to support close ones^a^ (%)	1,089	60.6	54.0	48.9	40.5	< 0.001	47.5	55.1	< 0.001	55.1	52.6	46.2	0.003	51.3
Not able to spend free time as wanted^a^ (%)	1,256	68.0	62.6	57.8	44.5	< 0.001	57.8	60.5	0.214	59.9	61.4	55.9	0.095	59.2
Not able to socialize as wanted^a^ (%)	1,303	70.0	62.8	59.2	53.2	< 0.001	58.1	64.7	0.002	63.2	63.9	56.8	0.011	61.4
Questing right choice in life^a^ (%)	795	49.3	40.4	34.5	24.3	< 0.001	38.4	36.5	0.361	43.6	37.6	31.6	< 0.001	37.4
**CBS Score** (median)	2,123	28.0	26.0	23.0	18.0	<.001^3^	24.0	25.0	.115^2^	28.0	24.0	22.0	<.001^3^	24,0
**CBS Subscale Material Burden** (median)	2,123	9.0	9.0	8.0	6.0	<.001^3^	8.0	8.0	.138^2^	10.0	8.0	7.0	<.001^3^	8.0
**CBS Subscale Psychosocial Burdens** (median)	2,123	18.0	16.0	15.0	11.0	<.001^3^	15.0	16.0	.007^2^	17.0	15.0	14.0	<.001^3^	15.0

^1^ Pearson Chi^2^-Test unless otherwise noted; ^2^ Mann-Whitney-U test; ^3^ Kruskal-Wallis test; ^a^ Includes the response categories: ‘agree somewhat, ‘agree mostly’ and ‘agree completely’; Gender: *M *Male, *F *Female; SSS: *L *Low, *M *Medium, *H *High

### Resources during the COVID-19 pandemic

Although most participants tried to live a normal daily life, they also used different coping strategies to adapt to their new, restricted living situation, such as taking more time to relax (42.7%) or spending more time in nature (44.6%). In addition, a third of the study participants increased the time they spent with their families and made more efforts to improve their health. There were some significant social differences, showing that older participants and those with low SSS were less likely to use most of the assessed coping strategies. A mixed pattern was noted regarding gender differences in coping strategies.

Almost half of the sample had high GSE, with a median of 29.0 and a mean (standard deviation) of 28.5 (5.7). However, large social differences in participants’ GSE were observed. For example, GSE scores were significantly higher among participants with higher educational attainment, higher SSS, and higher monthly net household income, as well as among male participants and older age groups (see Table 4, see Additional file 2, Supplementary Table 4_1 for the results stratified for immigration status, educational attainment (CASMIN) and monthly net household income).

**Table 4:** Resources during the COVID-19 pandemic differentiated by age, gender and SSS

Characteristics (*N* = 2,123)	*N*	Age					Gender			SSS				Total
		18–29	30–44	45–64	65–74	*p* ^1^	M	F	*p* ^1^	L	M	H	*p* ^1^	
**COVID-19 Pandemic related Resources and Coping Scale (CRCS)**														
Continue living normally^a^ (%)	1,760	80.0	82.0	84.0	85.4	0.478	83.8	81.9	0.306	79.8	82.8	85.9	0.015	82.9
Did things always wanted to do^a^ (%)	775	36.9	42.2	35.0	29.6	0.001	39.9	33.1	< 0.001	25.9	35.8	47.1	< 0.001	36.5
Focused on hobbies^a^ (%)	795	44.1	37.6	35.0	35.2	0.006	39.2	35.7	0.001	34.3	36.4	41.6	< 0.001	37.4
More in touch with family^a^ (%)	682	39.9	33.2	29.8	26.2	0.002	33.1	31.2	< 0.001	24.6	32.6	38.4	< 0.001	32.1
More in touch with friends^a^ (%)	512	27.1	26.8	22.5	19.6	0.125	25.0	23.3	0.001	18.0	23.6	30.3	< 0.001	24.1
Better care of personal health^a^ (%)	654	36.7	33.2	27.8	26.9	0.028	32.7	28.9	0.011	22.9	29.1	40.0	< 0.001	30.8
Personal activities online^a^ (%)	610	43.1	31.7	22.7	20.6	< 0.001	29.0	28.4	0.018	24.0	26.6	35.5	< 0.001	28.7
More time to spend with family^a^ (%)	763	41.9	43.4	31.9	25.2	< 0.001	37.6	34.3	0.003	23.2	37.8	45.5	< 0.001	35.9
Develop new routines^a^ (%)	606	34.7	30.9	26.3	22.3	0.004	31.6	25.4	< 0.001	18.3	29.0	37.4	< 0.001	28.5
More time in nature^a^ (%)	947	49.0	46.7	42.9	39.5	0.040	45.1	44.1	0.221	32.4	46.3	53.9	< 0.001	44.6
Time to pause^a^ (%)	884	45.1	40.7	42.4	36.5	0.237	42.7	40.5	0.030	33.3	42.0	48.8	< 0.001	41.6
Time to relax^a^ (%)	907	47.3	40.0	42.6	41.9	0.324	45.1	40.4	0.020	36.2	43.4	48.0	< 0.001	42.7
Help from others^a^ (%)	451	28.3	25.0	16.1	18.9	< 0.001	22.1	20.4	0.636	17.2	19.6	26.8	< 0.001	21.2
Supported others^a^ (%)	528	28.1	27.2	23.0	21.6	0.054	26.6	23.2	0.130	19.1	24.1	31.0	< 0.001	24.9
**CRCS score** (median)	2,123	37.0	36.0	34.0	34.0	<.001^3^	36.0	34.0	<.001^2^	32.0	35.0	37.0	<.001^3^	35.0
**CRCS Subscale Self-focused coping strategies** (median)	2,123	22.0	21.0	21.0	21.0	.002^3^	22.0	21.0	<.001^2^	19.0	22.0	23.0	<.001^3^	21.0
**CRCS Subscale Social engagement** (median)	2,123	15.0	14.0	13.0	13.0	<.001^3^	14.0	13.0	0.12^2^	12.0	13.0	14.0	<.001^3^	13.0
**General self-efficacy (GSE)**														
** High GSE** ^b^ (%)	1,044	42.1	42.2	53.0	61.1	< 0.001	52.2	46.1	0.005	32.5	50.7	62.6	< 0.001	49.2
** GSE** (median)	2,123	28.5	29.0	30.0	30.0	<.001^3^	30.0	29.0	.004^2^	27.0	30.0	30.0	<.001^3^	29.0
** GSE** (mean (Std. dev.)	2,123	27.6 (5.6)	28.0 (5.6)	28.7 (6.1)	29.9 (4.6)		28.8 (5.8)	28.2 (5.6)		26.3 (6.1)	28.7 (5.4)	30.2 (5.1)		28.5 (5.7)

^1^ Pearson Chi^2^-Test unless otherwise noted; ^2^ Mann-Whitney-U test; ^3^ Kruskal-Wallis test; ^a^ Includes the response categories: ‘agree completely’; ‘agree mostly’ and ‘somewhat agree’; ^b^ High GSE > = 29 [90]; Gender: *M *Male, *F *Female; SSS: *L *Low, *M *Medium, *H *High

### Self-rated health and mental well-being

Overall, 47.3% of the sample rated their health as very good or good, and 50.4% had a chronic disease. Older participants and participants with low SSS reported poorer self-rated health and were also more likely to have a chronic disease. For example, 73.6% of participants with low SSS rated their health as rather poor as opposed to just 31.2% of participants with high SSS.

Almost two-thirds of the sample reported high mental well-being levels, but there were notable differences by socioeconomic status, with significantly more participants with lower SSS reporting lower mental well-being levels. Contrary to self-rated health, younger participants (median = 14.0) rated their mental well-being lower than older participants (median = 17.0) (see Table 5, see Additional file 2 Supplementary Table 5_1 for the results stratified for immigration status, educational attainment (CASMIN) and monthly net household income).

**Table 5:** Self-rated health and mental well-being stratified by age, gender and SSS

Characteristics (*N* = 2,123)	*N*	Age					Gender			SSS				Total
		18–29	30–44	45–64	65–74	*p* ^1^	M	F	*p* ^1^	L	M	H	*p* ^1^	
**Self-rated health**						< 0.001			< 0.001				< 0.001	
Rather poor^a^	1,118	34.0	45.7	61.5	66.1		47.2	58.1		73.6	54.6	31.2		52.7
Rather good^b^	1,005	66.0	54.3	38.5	33.9		52.8	41.9		26.4	45.4	68.8		47.3
**Chronic disease**	1,070	28.3	36.9	62.7	71.1	< 0.001	43.8	57.1	< 0.001	70.0	46.3	37.2	< 0.001	50.4
**Mental well-being (WHO-5)**						< 0.001			< 0.001				< 0.001	
Low mental well-being^c^	804	40.4	40.9	39.9	22.9		31.4	44.4		56.4	38.1	20.6		37.9
High mental well-being^d^	1,319	59.6	59.1	60.1	77.1		68.6	55.6		43.6	61.9	79.4		62.1
Total score (median)	2,123	14.0	14.0	15.0	17.0	<.001^3^	16.0	14.0	<.001^2^	11.0	15.0	17.0	<.001^3^	15.0
Total score (mean (std. dev.))	2,123	13.3 (5.5)	13.3 (6.0)	13.6 (6.6)	15.7 (5.3)		14.6 (6.0)	13.0 (6.1)		10.8 (6.3)	13.9 (5.8)	16.4 (5.1)		13.8 (6.1)

^1^ Pearson Chi^2^-Test unless otherwise noted; ^2^ Mann-Whitney-U test; ^3^ Kruskal-Wallis test; ^a^ Includes the response categories: ‘satisfactory’, ‘poor’ and ‘very poor’; ^b^ Includes the response categories: ‘good’ and ‘very good’; ^c^ Total score below 13 [93]; ^d^ Total score of 13 or higher [93];

Gender: *M *Male, *F *Female; SSS: *L *Low, *M *Medium, *H *High

### Effects of socioeconomic and sociodemographic factors, resources, and burdens on self-rated health and mental well-being

Multivariable logistic regression analyses revealed that self-rated health was influenced by socioeconomic and sociodemographic factors as well as resources and burdens. Model 1 showed lower odds of participants assessing their self-rated health status as ‘rather good’ among older and female participants as well as among persons with lower SSS. People with high SSS were 4.4 times [3.30–5.81] more likely to have a ‘rather good’ self-rated health status than people with low SSS.

When resources and burdens were considered (model 2), GSE (1.10 [1.08–1.12]) increased the odds of a ‘rather good’ self-rated health status, while COVID-19-related burdens (CBS) had a negative effect (0.97 [0.96–0.98]). In model 3, only Psychosocial Burdens (0.97 [0.95–0.98]) reduced, while Self-Focused Coping Strategies (1.03 [1.00-1.05]) slightly increased the odds of reporting a ‘rather good’ self-rated health status. In the final model 4, the presence of a chronic disease was added and emerged as strong risk factor for self-rated health (0.19, [0.15–0.24]). The impact of socioeconomic and sociodemographic factors on self-rated health decreased when additional variables were included.

Model 1 significantly contributed to the prediction of self-rated health and explained 25% of its variance, which increased to 43% when additional variables were taken into account (model 4). All models had good model fit (Hosmer-Lemeshow-Test) (see Table 6).

**Table 6:** Multivariable analyses of determinants of self-rated health (binomial logistic regression, *N* = 1,962^1^)

Characteristics	Self-rated health^a^			
	Model 1 OR [CI 95%]	Model 2 OR [CI 95%]	Model 3 OR [CI 95%]	Model 4 OR [CI 95%]
**Constant**	0.958	0.249**	0.248**	0.369*
**Age**	0.97*** [0.96–0.98]	0.96*** [0.95–0.97)]	0.96*** [0.95–0.97]	0.97*** [0.96–0.98]
**Gender** (ref: male)	0.76** [0.62–0.92]	0.77* [0.62–0.94]	0.78* [0.63–0.96]	0.88 [0.71–1.11]
**Immigration status** (ref: without an immigration history)	1.05 [0.74–1.47]	0.99 [0.70–1.42]	0.99 [0.70–1.42]	0.84 [0.58–1.23]
**Educational attainment (CASMIN)** (ref: basic)				
Medium	1.48*** [1.17–1.86]	1.55*** [1.21–1.98]	1.57*** [1.23-2.00]	1.67*** [1.29–2.18]
Higher	1.75*** [1.29–2.38]	1.86*** [1.35–2.55]	1.87*** [1.36–2.58]	1.76*** [1.25–2.47]
**Monthly net household income** (ref: < 1,000 euros)				
1,000 to 2,999 euros	1.37 [0.97–1.92]	1.14 [0.80–1.63]	1.16 [0.81–1.66]	0.96 [0.65–1.40]
3,000 to 4,999 euros	2.05*** [1.41–2.97]	1.64* [1.11–2.41]	1.69** [1.14–2.50]	1.34 [0.89–2.04]
5,000 euros and more	1.78* [1.09–2.86]	1.39 [0.84–2.30]	1.41 [0.85–2.34]	1.01 [0.59–1.73]
**SSS** (ref: low)				
Medium	1.90*** [1.47–2.43]	1.46** [1.12–1.90]	1.46** [1.12–1.91]	1.14 [0.85–1.51]
High	4.38*** [3.30–5.81]	3.01*** [2.23–4.06]	3.04*** [2.25–4.11]	2.57*** [1.86–3.54]
**GSE score**		1.10*** [1.08–1.12]	1.10*** [1.08–1.12]	1.09*** [1.07–1.12]
**CRCS score**		1.01 [1.00-1.02]		
**CBS score**		0.97*** [0.96–0.98]		
**CRCS Subscale Self-focused coping strategies**			1.03* [1.00-1.05]	1.03* [1.00-1.05]
**CRCS Subscale Social engagement**			0.98 [0.95–1.02]	0.99 [0.95–1.02]
**CBS Subscale Material Burden**			0.98 [0.95-1.00]	0.96** [0.94–0.99]
**CBS Subscale Psychosocial Burden**			0.97*** [0.95–0.98]	0.98* [0.96-1.00]
**Chronic disease** (ref: no)				0.19*** [0.15–0.24]
**Hosmer-Lemeshow-Test ** **(** ***p*** **-value)**	0.56	0.81	0.90	0.62
**Nagelkerkes R** ^**2**^	0.25	0.32	0.33	0.43

^1^Sample reduced due to missing values in the variable monthly net household income. ^a^The dependent variable ‘self-rated health’ is coded so that 0= ‘rather poor’ and 1= ‘rather good’ (Includes the response categories: ‘good’ and ‘very good’.). *GSE *General self-efficacy, *CRCS *COVID-19 Pandemic related Resources and Coping Scale, *CBS *COVID-19 Pandemic-related Burden Scale. *p < = 0.05; **p < = 0.01; ***p < = 0.001

Similar results were found in the analysis of mental well-being (see Table 7). Model 1 showed that the odds of having a high mental well-being level was higher for people with a higher level of education (1.45 [1.06–1.99]), medium (1.93 [1.53–2.44]) or high SSS (4.13 [3.11–5.48]), as well as for older participants (1.02 [1.01–1.02]); lower odds were observed for female participants (0.64 [0.53–0.78]). Resources increased and burdens decreased the odds of high mental well-being (models 2 and 3), with the exception of the Material Burden Subscale of the CBS. In the final model 4, having a chronic disease was associated with 53% lower odds of high mental well-being. Immigration status did not emerge as a significant predictor of neither self-rated health nor mental well-being (see Tables 6 and 7) in any of the models. The contribution of social determinants toward a high mental well-being level decreased from model 1 to model 4, but only medium SSS became insignificant.

The logistic regression models were statistically significant and had good model fit (Hosmer-Lemeshow-Test). The explained variance increased from 16% in model 1 to 34% in model 4 (see Table 7).

**Table 7:** Multivariable analyses of determinants of mental well-being (binomial logistic regression, *N* = 1,962^1^)

Characteristics	Mental well-being^a^			
	Model 1 OR [CI 95%]	Model 2 OR [CI 95%]	Model 3 OR [CI 95%]	Model 4 OR [CI 95%]
**Constant**	0.299***	0.024***	0.020***	0.024***
**Age**	1.02*** [1.01–1.02]	1.01* [1.00-1.02]	1.01* [1.00-1.02]	1.02*** [1.01–1.03]
**Gender** (ref: male)	0.64*** [0.53–0.78]	0.63*** [0.51–0.78]	0.71** [0.57–0.88]	0.76* [0.61–0.94]
**Immigration status ** (ref: without an immigration history)	0.92 [0.66–1.28]	0.87 [0.61–1.25]	0.81 [0.57–1.16]	0.76 [0.52–1.09]
**Educational attainment (CASMIN)** (ref: basic)				
Medium	1.02 [0.82–1.28]	1.03 [0.81–1.31]	1.07 [0.84–1.37]	1.08 [0.84–1.39]
Higher	1.45* [1.06–1.99]	1.55* [1.10–2.17]	1.52* [1.08–2.14]	1.44* [1.02–2.03]
**Monthly net household income** (ref: < 1,000 euros)				
1,000 to 2,999 euros	1.58** [1.17–2.14]	1.28 [0.92–1.77]	1.42* [1.02–1.99]	1.33 [0.95–1.86]
3,000 to 4,999 euros	1.52* [1.08–2.14]	1.12 [0.77–1.62]	1.31 [0.90–1.92]	1.18 [0.80–1.74]
5,000 euros and more	2.06** [1.27–3.36]	1.50 [0.88–2.54]	1.70 [1.00-2.89]	1.47 [0.86–2.50]
**SSS** (ref: low)				
Medium	1.93*** [1.53–2.44]	1.31* [1.02–1.70]	1.37* [1.06–1.77]	1.23 [0.94–1.60]
High	4.13*** [3.11–5.48]	2.36*** [1.73–3.22]	2.41*** [1.77–3.29]	2.17*** [1.58–2.98]
**GSE score**		1.11*** [1.09–1.14]	1.12*** [1.10–1.15]	1.12*** [1.09–1.14]
**CRCS score**		1.05*** [1.04–1.06]		
**CBS score**		0.96*** [0.95–0.97]		
**CRCS Subscale Self-focused coping strategies**			1.05*** [1.02–1.07]	1.05*** [1.02–1.07]
**CRCS Subscale Social engagement**			1.04* [1.01–1.08]	1.04* [1.01–1.08]
**CBS Subscale Material Burden**			1.02 [0.99–1.04]	1.01 [0.99–1.04]
**CBS Subscale Psychosocial Burden**			0.92*** [0.90–0.94]	0.92*** [0.90–0.94]
**Chronic disease** (ref: no)				0.47*** [0.37–0.60]
**Hosmer-Lemeshow-Test ** **(** ***p*** **-value)**	0.92	0.90	0.10	0.84
**Nagelkerkes R** ^**2**^	0.16	0.31	0.32	0.34

^1^Sample reduced due to missing values in the variable monthly net household income. ^a^The dependent variable ‘mental well-being’ is coded so that 0= ‘low’ and 1= ‘high’ (Total score of 13 or higher [93]). *GSE *General self-efficacy, *CRCS *COVID-19 Pandemic related Resources and Coping Scale, *CBS *COVID-19 Pandemic-related Burden Scale. *p < = 0.05; **p < = 0.01; ***p < = 0.001

## Discussion

The aim of the study was to explore the complex associations between socioeconomic and sociodemographic factors, changes in life circumstances, pandemic-related burdens and resources, and indicators of self-rated health and well-being among adults living in Germany. As such, the study contributes to a better understanding of how endemic health inequities, which have been documented in numerous studies both internationally [37, 38] and in Germany [39–42] contributed to the adverse impacts of the COVID-19 pandemic, which has been characterized as a syndemic pandemic [48–54]. The main strength of our study lies in utilizing multiple measures of socioeconomic status, sociodemographic differences, and living conditions during the COVID-19 pandemic, and leveraging them to characterize life experiences and their influence on self-rated health and mental well-being. Another strength of the study is that it was carried out at the end of the COVID-19 pandemic, providing a retrospective assessment of the whole pandemic period and all the associated changes in participants’ social circumstances and health.

### Research hypothesis 1

Our findings support hypothesis 1 that participants with a lower socioeconomic status had poorer self-rated health and lower mental well-being during the COVID-19 pandemic. Overall, 52.7% of the participants rated their overall health status as rather poor, which was much more common among socioeconomically disadvantaged groups. This was also shown, for example, for educational attainment in a representative sample from Germany [94]. Our findings are also aligned with previous research indicating that people with higher educational attainment [8, 11, 27, 29] or higher household income [13] were more likely to report higher levels of mental well-being.

### Research hypothesis 2

Our hypothesis that poorer self-rated health and well-being were associated with older age, female gender, and immigration status was only partially supported by the data. In our sample, men and younger people were significantly more likely to report better self-rated health, as also shown in other studies conducted both prior to and during the COVID-19 pandemic [5, 23]. Similar to prior research [6, 8, 10, 13, 22, 26–28], higher mental well-being was reported by men in our sample. However, contrary to our assumptions, older participants in our sample reported higher levels of well-being, a finding common to other studies [6, 8, 11, 13, 22]. This could potentially be explained by the dramatic changes in life circumstances experienced by younger demographics and their disproportionate toll on mental well-being. Interestingly, this study found almost no significant differences for immigration status, which could be due to the very similar socioeconomic status of the study participants, regardless of their immigration status.

### Research hypothesis 3

As stated in hypothesis 3, socioeconomic differences were identified in the extent to which participants experienced life changes and burdens associated with PHSM, supporting a better understanding of the possible mechanism of a syndemic pandemic [48, 49, 54, 55, 64, 70]. Overall, 39.8% of the participants in our sample experienced changes in their personal and professional life due to PHSM. Contrary to hypotheses 3 and prior research findings [31, 32], however, life changes associated with PHSM were reported more frequently by participants with higher socioeconomic status. An exception was loss of employment [33, 34], which was significantly more common among participants with low SSS and among the lowest income group in our study. In addition, younger and female participants, as well as participants with lower SSS reported more long-term life changes and burdens (see CBS scale) associated with PHSM. This might be associated with lower resilience and ability to cope with specific restrictions due to the greater impact of these restrictions among the more socioeconomically disadvantaged groups [55]. These findings may provide a valid reflection of the long-term effects of PHSM and pre-existing social disparities. However, our retrospective assessment at the end of the COVID-19 pandemic may have masked potentially stronger socioeconomic differences resulting from PHSM during the COVID-19 pandemic, thus underestimating their impact on participants’ pandemic experiences. Research hypothesis 3 is thus only partially supported by the data, highlighting the importance of distinguishing between short and long-term pandemic effects, as well as concurrent and retrospective assessments [74].

### Research hypothesis 4

Resources increase the ability to cope with challenging life situations and are associated with better health outcomes, but their unequal distribution contributes to health inequities [71–73]. The higher level of burdens experienced by participants with lower SSS during the pandemic goes hand in hand with disparities in access to coping resources, as measured by the GSE and the CRCS scale. Higher scores on the GSE as well as the global CRCS scale and the two CRCS subscales were reported by participants with higher socioeconomic status, which provides empirical support for hypothesis 4. In contrast, the limited access to such resources among participants with low SSS emerged as a significant driver of health inequity in our study. Prior studies conducted in Germany have identified an association between coping style and mental well-being [35]. Individuals with higher psychological well-being dedicated more time to their family and friends or used more time for themselves [36]. In contrast, individuals with a lower psychological well-being used significantly more strategies, such as entertainment at home [36]. However, prior research did not assess socioeconomic and sociodemographic differences in coping styles; our findings thus provide further insight into the contribution of resources and coping mechanisms to health and well-being during the COVID-19 pandemic.

#### Research hypothesis 5

The logistic regression analysis revealed a social gradient in the impact of educational attainment (CASMIN), monthly net household income, and SSS on both self-rated health and mental well-being. Regarding sociodemographic differences, the results were inconsistent. Recognizing that participants with lower socioeconomic status had higher burdens (CBS score) and lower resources (GSE, CRCS score), differences in exposure and vulnerability [64, 66], as well as limited access to resources [72, 73], have been proposed as explanations for health inequities prior to the pandemic and may also be considered as drivers of disparities in health and mental well-being during the COVID-19 pandemic. Accordingly, hypothesis 5 is supported by the data.

Overall, the results of our study support the syndemic nature of the COVID-19 pandemic [48–54], as socioeconomically disadvantaged populations experienced higher levels of burdens and had fewer resources to cope with changes in social life as well as with the higher prevalence of chronic diseases in this population, which can be seen as an indicator of endemic disparities [49, 64, 66, 74]. Our findings can be easily integrated into the adapted model of CSDH during the COVID-19 pandemic [70]. Specifically, our results highlight the importance of both structural (e.g., socioeconomic factors) and intermediary (e.g., resources and burdens) determinants of health inequity [47]. Interestingly, although we found fewer changes in social life due to PHSM among participants with lower socioeconomic status, the burden and long-term effects associated with these changes are higher in this group [55]. Differences in PHSM-driven changes experienced across socioeconomic groups are typically related to inequitable employment structures, such as working conditions or job insecurity. Working from home, for example, is much more accessible to employees with higher occupational status. Participants with low socioeconomic status are, therefore, less likely to work from home, which also exposes them to an increased risk of COVID-19 infection [48, 55].

Overall, our findings suggest that the quality, not the quantity of pandemic-related life changes had a more significant impact on participants’ health and mental well-being. Accordingly, even if participants with higher socioeconomic status experienced more types of life changes during the pandemic, some of these changes may have opened up new opportunities (e.g., spending more time with family, not commuting) or could be more easily managed if sufficient resources were available [72]. This is also supported by the fact that, in our study, significantly more people with low socioeconomic status reported that their livelihood was threatened or that it would take them a long time to regain their pre-pandemic living standard.

### Limitations

Some limitations of this study need to be mentioned. First, this was an online cross-sectional survey, which could have limited the representativeness of our data. However, the use of a quota sampling procedure ensured that our sample was representative of the gender, age, and federal state distribution of the general population in Germany, based on Eurostat 2020 data. In addition, by setting a quota of 30% for participants with low educational attainment, we were able to conduct adequately powered statistical analyses for this group. The proportion of immigrants and their (direct) descendants and their often lower socioeconomic background [95, 96] in Germany is not reflected in our sample. Since in our study immigrants and their (direct) descendants did not differ from those without an immigration history with regard to their socioeconomic status, our findings offer valuable insight into the precedence of socioeconomic status over immigration status in shaping health inequity in Germany during the COVID-19 pandemic. In addition to lower-income segments of the immigrant population, people who identify as a gender other than male or female may have also been missed, since gender was assessed as a binary variable. Another limitation to the generalizability of our findings to the general population is that the participants were members of an online panel, so people without access to the Internet or sufficient digital skills are not represented in the study sample. In addition, our sample consists of individuals willing to participate in online research, which may introduce a self-selection bias.

Second, the methodology of a cross-sectional study does not allow for causal statements. Thirdly, the data were collected toward the end of the COVID-19 pandemic PHSM enforcement and although they allow for an overall assessment, specific changes that occurred over the course of the pandemic cannot be tracked. Fourth, significant societal changes and pressures emerged in Germany in 2022 due to the war in Ukraine, which may have influenced participants’ responses to the study questions, particularly regarding the assessment of their mental well-being. Nevertheless, because the questions explicitly referred to the COVID-19 pandemic, it is assumed that these responses represent the actual pandemic-related changes experienced by the participants.

## Conclusion

The syndemic pandemic concept highlights the complexity of health inequities under conditions of major social challenges of national and international proportion, such as those created by the COVID-19 pandemic. Therefore, assessing the consequences of COVID-19-related social restrictions is of great importance beyond the scope of this pandemic, as it informs future preparedness efforts aimed at reducing social and health disparities.

Our findings contribute to a better, more granular understanding of differences in vulnerability associated with social circumstances, resulting burdens, and available coping strategies. The data also reveal patterns of social and health inequity that should be further explored using other analytical techniques such as cluster analysis. Identifying groups with similar pandemic experiences will arguably allow for a clearer description of which intersections of socioeconomic and sociodemographic variables are associated with better or worse health outcomes. In addition, detailed analyses of the health inequities experienced by people living with chronic diseases are needed to better understand this pathway of the syndemic pandemic model.

Our findings show that self-rated health and mental well-being are affected by both structural and intermediary determinants in the context of a pandemic, which calls for different strategies to promote health equity. In addition to financial support in the event of income loss (e.g., reduced work hour benefits), other systemic measures should be taken to bolster resilience in groups with low socioeconomic status in the long run. This could include the provision of psychological, educational, and social support to cope with critical situations. At the same time, efforts should be made to ensure that all neighborhoods are conducive to healthy living, for example by providing safe infrastructure and sufficient green spaces and outdoor play areas.

The large social gradient identified in our study suggests that, even after having overcome the state of emergency caused by COVID-19 pandemic, we need to be aware that not all population groups have recovered economically and returned to pre-pandemic living standards. These life changes and experiences need to be incorporated into health promotion strategies to support socioeconomically disadvantaged groups during and beyond critical times in history. In addition, health promotion interventions should prioritize strengthening coping resources, given their limited availability among socioeconomically disadvantaged groups.

In conclusion, we recommend that future research implement a thorough and differentiated assessment of social living conditions, health-related resources, and risks during health crises to identify and monitor their effects on different population groups. This approach will not only enhance our understanding of syndemic pandemic impacts but also provide insights for tailoring public health interventions. In addition to initiatives aimed at combating the spread and health consequences of SARS-CoV-2, health promotion measures should be an integral part of crisis management. Given their prominence as risk factors in our study, policymakers and healthcare providers should systematically incorporate strategies that effectively address markers of socioeconomic disadvantage to reduce health disparities during future health crises.

## Supplementary Information

Supplementary Material 1. Items on the CBS and CRCS scales. Information is provided on the items and components of the CBS and CRCS scales and subscales.

Supplementary Material 2. Additional analyses are provided regarding immigration status, educational attainment (CASMIN) and monthly net household income. Findings are presented regarding immigration status, educational attainment (CASMIN) and monthly net household income.

## Data Availability

The data that support the findings of this paper are available from the corresponding author upon reasonable request.

## Abbreviations

CBS: COVID-19 Pandemic related Burden Scale
CRCS: COVID-19 Pandemic-Related Resources and Coping Scale
F: Female
GSE: General self-efficacy
ID: Immigrants and their (direct) descendants
M: Male
NIH: No immigration history
PHSM: Public health and social measures
SSS: Subjective social status
