# Supplementary material for: Socioeconomic and sociodemographic differences in the consequences of the COVID-19 pandemic and their impact on self-rated health and mental well-being: results from a cross-sectional study in Germany
Source: BMC Public Health. 2025 Jul 22;25:2523. doi: 10.1186/s12889-025-23698-w (PMC12281828; doi:10.1186/s12889-025-23698-w)
Supplement: Supplementary file 1 — Supplementary Material 1. Items on the CBS and CRCS scales. Information is provided on the items and components of the CBS and CRCS scales and subscales. [file 12889_2025_23698_MOESM1_ESM.docx]

**Socioeconomic and sociodemographic differences in the consequences of the COVID-19 pandemic and their impact on self-rated health and mental well-being. Results from a cross-sectional study in Germany.**

Babitsch Birgit^1*^, Ciupitu-Plath, Cristina^2^

^1^ Department of New Public Health, Institute of Health Research and Education, School of Human Sciences, Osnabrück University, Osnabrück, Germany

^2^ Department of Public Health, Bastyr University, Kenmore, Washington, USA

*** Correspondence:** Birgit Babitsch

bbabitsch@uos.de

**Supplementary Material**

## File name: Additional file 1

## File format including the three-letter file extension: pdf-Document

## Title: Items on the CBS and CRCS scales

## Description of data: Information is provided on the items and components of the CBS and CRCS scales and subscales.

Supplementary Table 1 COVID-19 Pandemic related Burden Scale (CBS)

To estimate the overall burden, the CBS score was calculated by adding all remaining 12 items (range 12-60). Reliability was good, with Cronbach’s alpha = .869.

Principal component analysis (PCA) revealed two independent factors of the CBS: 1) material burden and 2) psychosocial burden. One item was excluded due to weak discrimination between the two factors. Separate scores for the subscales ‘Material burden’ (range: 6-30, Cronbach’s alpha =.833) and ‘Psychosocial burden’ (range: 6-30, Cronbach’s alpha = .851) were calculated.

| **CBS items** | **Shortform** | **CBS Subscale Material Burden** | **CBS Subscale Psychosocial Burden** |
| --- | --- | --- | --- |
| My livelihood is threatened (e.g., due to loss of employment). | Livelihood threatened |  |  |
| Even after the COVID-19 pandemic is over, it will take me a long time to. | Long time to get back to previous standard of living |  |  |
| My housing situation has worsened considerably. | Housing situation worsened |  |  |
| I had to apply for state benefits. | Applied for state benefits |  |  |
| I had to give up my own business. | Gave up own business |  |  |
| I lost the opportunity to reach my full potential at work. | Not reaching full potential at work |  |  |
| I felt socially isolated. | Felt socially isolated |  |  |
| I was very exhausted due to the multiple challenges resulting from the COVID-19 pandemic containment measures. | Exhausted due to the multiple challenges |  |  |
| I no longer felt part of a social group. | Not part of a social group |  |  |
| It was challenging for me not to be able to support my close ones as much as I would have liked to. | Not able to support close ones |  |  |
| It was very challenging for me time the way I wanted to. | Not able to spend free time as wanted |  |  |
| It was very challenging for me not to be able to socialize the way I wanted to. | Not able to socialize as wanted |  |  |
| My experience with the COVID-19 pandemic has made me question whether I have made the right choices in life. | Questioning life choices | *not included* | *not included* |

Supplementary Table 2 COVID-19 Pandemic-Related Resources and Coping Scale (CRCS)

To estimate the overall level of coping, the CRCS score was calculated by adding all 12 items (range 12-60). Reliability was good with Cronbach’s alpha for standardized items = .881.

For the CRCS, PCA was performed, identifying two factors: 1) self-focused coping strategies and 2) social engagement. Two items were excluded due to weak discrimination between the two factors. Separate scores for the subscales ‘Self-focused coping strategies’ (range: 7-35, Cronbach’s alpha = .856) and ‘Social engagement’ (range: 5-25, Cronbach’s alpha = .756) were calculated.

| **CRCS items** | **Shortform** | **CRCS Subscale Self-focused coping strategies** | **CRCS Subscale Social engagement** |
| --- | --- | --- | --- |
| I tried to organise my daily life as normally as possible. | Continue living normally | *not included* | *not included* |
| I did things I had always wanted to do (e.g., redecorate). | Did things always wanted to do |  |  |
| I focused more on my hobbies. | Focused on hobbies |  |  |
| I stayed in touch with my family more often than usual. | More in touch with family |  |  |
| I stayed in touch with my friends more often than usual. | More in touch with friends |  |  |
| I took better care of my health (e.g., by exercising). | Better care of personal health |  |  |
| I started doing many of my personal activities online (e.g., socialising, sports classes). | Personal activities online |  |  |
| Because of all the changes, I had more time to spend with my family. | More time to spend with family | *not included* | *not included* |
| It helped me to develop new routines in my everyday life. | Develop new routines |  |  |
| I spent much more time in nature. | More time in nature. |  |  |
| I enjoyed having time to pause. | Time to pause |  |  |
| - I took more time to relax. | Time to relax |  |  |
| I got help from other people. | Help from others |  |  |
| I engaged in service to other people to support them during the COVID-19 pandemic. | Supported others |  |  |
