# Supplementary material for: Socioeconomic and sociodemographic differences in the consequences of the COVID-19 pandemic and their impact on self-rated health and mental well-being: results from a cross-sectional study in Germany
Source: BMC Public Health. 2025 Jul 22;25:2523. doi: 10.1186/s12889-025-23698-w (PMC12281828; doi:10.1186/s12889-025-23698-w)
Supplement: Supplementary file 2 — Supplementary Material 2. Additional analyses are provided regarding immigration status, educational attainment (CASMIN) and monthly net household income. Findings are presented regarding immigration status, educational attainment (CASMIN) and monthly net household income. [file 12889_2025_23698_MOESM2_ESM.docx]

**Socioeconomic and sociodemographic differences in the consequences of the COVID-19 pandemic and their impact on self-rated health and mental well-being. Results from a cross-sectional study in Germany.**

Babitsch Birgit^1*^, Ciupitu-Plath, Cristina^2^

^1^ Department of New Public Health, Institute of Health Research and Education, School of Human Sciences, Osnabrück University, Osnabrück, Germany

^2^ Department of Public Health, Bastyr University, Kenmore, Washington, USA

*** Correspondence:** Birgit Babitsch

bbabitsch@uos.de

**Supplementary Material**

## File name: Additional file 2

## File format including the three-letter file extension: pdf-Document

## Title: Additional analyses are provided regarding immigration status, educational attainment (CASMIN) and monthly net household income

## Description of data: Findings are presented regarding immigration status, educational attainment (CASMIN) and monthly net household income.

Supplementary Table 2_1. Experiences and changes in the life situation through the COVID-19 pandemic stratified by immigration status, educational attainment (CASMIN) and monthly net household income

| **Characteristics (N=2,123)** | **N** | **Immigration status** | | | **Educational attainment (CASMIN)** | | | | **Monthly net household income** | | | | | | **Total** |
| --- | --- | --- | --- | --- | --- | --- | --- | --- | --- | --- | --- | --- | --- | --- | --- |
|  |  | **NIH** | **ID** | **p^1^** | **B** | **M** | **H** | **p^1^** | **up to under 1,000 euros** | **1,000 to 2,999 euros** | **3,000 to 4,999 euros** | **5,000 euros and more** | **No ans-wer** | **p^1^** |  |
| **Changes in life through measures to contain the COVID-19 pandemic** |  |  |  | .162 |  |  |  | .035 |  |  |  |  |  | .085 |  |
| Rather strong^a^ (%) | 1,115 | 52.7 | 50.8 |  | 48.6 | 53.8 | 55.5 |  | 50.6 | 52.0 | 54.7 | 48.5 | 55.9 |  | 52.5 |
| **PHSM** |  |  |  |  |  |  |  |  |  |  |  |  |  |  |  |
| **Working from home** (%) |  |  |  | .400 |  |  |  | <.001 |  |  |  |  |  | <.001 |  |
| Yes | 644 | 30.0 | 33.2 |  | 8.6 | 29.8 | 64.0 |  | 14.4 | 23.0 | 36.8 | 64.8 | 32.3 |  | 30.3 |
| No | 606 | 28.4 | 30.2 |  | 32.8 | 30.7 | 17.3 |  | 23.5 | 29.9 | 33.2 | 21.4 | 20.5 |  | 28.5 |
| Does not apply to me | 873 | 41.6 | 36.7 |  | 58.6 | 39.5 | 18.7 |  | 62.1 | 47.1 | 30.0 | 13.8 | 47.2 |  | 41.1 |
| **Extra work** (%) |  |  |  | .617 |  |  |  | <.001 |  |  |  |  |  | <.001 |  |
| Yes | 408 | 19.2 | 19.6 |  | 13.4 | 19.5 | 27.2 |  | 8.6 | 17.2 | 24.7 | 31.6 | 12.4 |  | 19.2 |
| No | 945 | 44.2 | 47.2 |  | 35.6 | 46.8 | 52.6 |  | 30.0 | 42.1 | 51.2 | 55.1 | 43.5 |  | 44.5 |
| Does not apply to me | 770 | 36.6 | 33.2 |  | 51.0 | 33.7 | 20.2 |  | 61.3 | 40.7 | 24.1 | 13.3 | 44.1 |  | 36.3 |
| **Loss of employment** (%) |  |  |  | .571 |  |  |  | <.001 |  |  |  |  |  | <.001 |  |
| Yes | 240 | 11.1 | 13.6 |  | 9.8 | 11.7 | 12.6 |  | 13.2 | 10.9 | 11.1 | 11.2 | 11.8 |  | 11.3 |
| No | 1,116 | 52.7 | 51.3 |  | 41.8 | 53.8 | 65.8 |  | 28.8 | 48.8 | 64.8 | 70.9 | 44.7 |  | 52.6 |
| Does not apply to me | 767 | 36.2 | 35.2 |  | 48.4 | 34.5 | 21.6 |  | 58.0 | 40.3 | 24.1 | 17.9 | 43.5 |  | 36.1 |
| **Work hour reductions** (%) |  |  |  | .443 |  |  |  | <.001 |  |  |  |  |  | <.001 |  |
| Yes | 321 | 14.8 | 18.1 |  | 14.1 | 15.7 | 15.3 |  | 10.3 | 14.0 | 19.3 | 16.8 | 11.8 |  | 15.1 |
| No | 1,016 | 47.9 | 47.2 |  | 36.4 | 49.5 | 61.3 |  | 30.5 | 44.1 | 56.9 | 66.3 | 41.0 |  | 47.9 |
| Does not apply to me | 786 | 37.3 | 34.7 |  | 49.5 | 34.8 | 23.4 |  | 59.3 | 41.8 | 23.8 | 16.8 | 47.2 |  | 37.0 |
| **Reduction overtime and/or holiday** (%) |  |  |  | .427 |  |  |  | <.001 |  |  |  |  |  | <.001 |  |
| Yes | 317 | 14.9 | 15.6 |  | 10.7 | 14.5 | 22.2 |  | 4.1 | 14.3 | 19.3 | 21.9 | 11.2 |  | 14.9 |
| No | 1,023 | 47.8 | 51.8 |  | 39.2 | 50.9 | 55.3 |  | 32.5 | 44.0 | 57.5 | 62.8 | 45.3 |  | 48.2 |
| Does not apply to me | 783 | 37.3 | 32.7 |  | 50.1 | 34.6 | 22.5 |  | 63.4 | 41.7 | 23.3 | 15.3 | 43.5 |  | 36.9 |
| **Delegation of other tasks** (%) |  |  |  | .148 |  |  |  | <.001 |  |  |  |  |  | <.001 |  |
| Yes | 328 | 15.0 | 20.1 |  | 8.3 | 16.5 | 23.8 |  | 5.8 | 12.6 | 21.2 | 24.5 | 15.5 |  | 15.4 |
| No | 1,020 | 48.2 | 46.7 |  | 42.0 | 49.6 | 53.7 |  | 32.5 | 46.7 | 55.2 | 59.2 | 40.4 |  | 48.0 |
| Does not apply to me | 775 | 36.9 | 33.2 |  | 49.8 | 34.0 | 22.5 |  | 61.7 | 40.8 | 23.6 | 16.3 | 44.1 |  | 36.5 |
| **Homeschooling** (%) |  |  |  | .764 |  |  |  | <.001 |  |  |  |  |  | <.001 |  |
| Yes | 400 | 18.7 | 20.6 |  | 14.6 | 18.6 | 25.8 |  | 7.8 | 13.2 | 26.6 | 35.7 | 20.5 |  | 18.8 |
| No | 787 | 37.1 | 37.2 |  | 32.8 | 38.2 | 40.9 |  | 28.4 | 36.3 | 41.1 | 44.4 | 31.1 |  | 37.1 |
| Does not apply to me | 936 | 44.3 | 42.2 |  | 52.6 | 43.2 | 33.3 |  | 63.8 | 50.5 | 32.3 | 19.9 | 48.4 |  | 44.1 |
| **Perceived threat from the COVID-19 pandemic** |  |  |  | .120 |  |  |  | .662 |  |  |  |  |  | .784 |  |
| Rather strong^a^ (%) | 855 | 40.1 | 41.7 |  | 40.8 | 39.9 | 40.4 |  | 40.7 | 40.9 | 39.1 | 38.8 | 42.2 |  | 40.3 |
| **Perceived burden of the COVID-19 pandemic** |  |  |  | .399 |  |  |  | .195 |  |  |  |  |  | .615 |  |
| Rather strong^a^ (%) | 1,057 | 49.5 | 52.3 |  | 48.7 | 51.4 | 47.6 |  | 51.4 | 51.3 | 48.4 | 46.4 | 47.2 |  | 49.8 |
| **Perceived restrictions due to the COVID-19 pandemic** |  |  |  | .282 |  |  |  | .323 |  |  |  |  |  | .332 |  |
| Rather strong^a^ (%) | 1,092 | 50.9 | 56.8 |  | 48.7 | 52.9 | 52.1 |  | 51.0 | 51.6 | 49.8 | 50.0 | 58.4 |  | 51.4 |
| **Overall assessment of dealing with the COVID-19 pandemic** |  |  |  | .022 |  |  |  | .095 |  |  |  |  |  | .272 |  |
| Rather difficult^b^  (%) | 759 | 35.7 | 36.2 |  | 36.5 | 37.5 | 30.6 |  | 35.0 | 37.2 | 35.4 | 28.1 | 39.1 |  | 35.8 |
| **Change in living situation due to the relaxation of COVID-19-related restrictions** |  |  |  | .637 |  |  |  | <.001 |  |  |  |  |  | <.001 |  |
| Rather strong^a^ (%) | 919 | 43.2 | 43.7 |  | 34.9 | 45.9 | 49.9 |  | 33.7 | 40.4 | 48.8 | 54.1 | 41.6 |  | 43.3 |
| **Percentage of adjustment between current living situation and everyday life before the coronavirus pandemic** |  |  |  | .080^2^ |  |  |  | .075^3^ |  |  |  |  |  | <.001^3^ |  |
| Median | 2,123 | 79.0 | 71.0 |  | 75.0 | 80.0 | 77.0 |  | 65.0 | 77.0 | 80.0 | 80.0 | 72.0 |  | 79.0 |

^1^ Pearson Chi^2^-Test unless otherwise noted; ^2^ Mann-Whitney-U test; ^3^ Kruskal-Wallis test; ^a^ Includes the response categories: ‘rather strong’ and ‘very strong’; ^b^ Includes the response categories: ‘difficult’ and ‘very difficult’; Immigration status: NIH: No immigration history, ID: Immigrants and their (direct) descendants; Educational attainment (CASMIN): B: Basic, M: Medium, H: Higher

Supplementary Table 3_1 Experienced burdens measured by the COVID-19 Pandemic-related Burden Scale (CBS) differentiated by immigration status, educational attainment (CASMIN) and monthly net household income

| **Characteristics (N=2,123)** | **N** | **Immigration status** | | | **Educational attainment (CASMIN)** | | | | **Monthly net household income** | | | | | | **Total** |
| --- | --- | --- | --- | --- | --- | --- | --- | --- | --- | --- | --- | --- | --- | --- | --- |
|  |  | **NIH** | **ID** | **p^1^** | **B** | **M** | **H** | **p^1^** | **up to under 1,000 euros** | **1,000 to 2,999 euros** | **3,000 4,999 euros** | **5,000 euros and more** | **No ans-wer** | **p^1^** |  |
| Livelihood threatened^a^ (%) | 477 | 22.1 | 26.1 | .193 | 24.2 | 20.7 | 23.8 | .184 | 33.7 | 22.7 | 17.5 | 19.9 | 24.8 | <.001 | 22.5 |
| Long time to get back to previous standard of living^a^ (%) | 728 | 33.3 | 43.7 | .003 | 37.9 | 33.7 | 30.3 | .029 | 44.9 | 37.8 | 27.3 | 20.4 | 39.8 | <.001 | 34.3 |
| Housing situation worsened^a^ (%) | 368 | 16.4 | 26.6 | <.001 | 18.6 | 15.4 | 19.8 | .071 | 24.7 | 16.9 | 15.3 | 18.9 | 14.3 | .015 | 17.3 |
| Applied for state benefits^a^ (%) | 428 | 19.6 | 25.1 | .067 | 23.3 | 17.6 | 21.3 | .013 | 42.8 | 19.7 | 13.0 | 18.9 | 15.5 | <.001 | 20.2 |
| Gave up own business^a^ (%) | 238 | 10.3 | 20.1 | <.001 | 9.9 | 9.5 | 17.1 | <.001 | 15.6 | 9.2 | 10.6 | 18.9 | 9.3 | <.001 | 11.2 |
| Not reaching full potential at work^a^ (%) | 424 | 19.3 | 26.6 | .014 | 18.6 | 19.5 | 22.9 | .195 | 23.5 | 20.3 | 18.4 | 18.9 | 19.9 | .569 | 20.0 |
| Felt socially isolated^a^ (%) | 880 | 41.0 | 46.2 | .150 | 38.9 | 41.7 | 44.7 | .158 | 52.3 | 40.9 | 39.4 | 39.8 | 37.9 | .008 | 41.5 |
| Exhausted due to the multiple challenges^a^ (%) | 913 | 42.7 | 45.7 | .415 | 40.5 | 44.9 | 42.5 | .189 | 44.0 | 43.7 | 42.9 | 38.8 | 42.9 | .784 | 43.0 |
| Not part of a social group^a^ (%) | 834 | 38.9 | 43.2 | .233 | 40.5 | 38.2 | 40.0 | .615 | 48.1 | 40.3 | 35.8 | 37.2 | 34.8 | .011 | 39.3 |
| Not able to support close ones^a^ (%) | 1,089 | 50.9 | 54.8 | .302 | 52.3 | 52.3 | 47.4 | .183 | 52.3 | 53.0 | 49.7 | 46.4 | 51.6 | .450 | 51.3 |
| Not able to spend free time as wanted^a^ (%) | 1,256 | 59.1 | 59.3 | .968 | 57.4 | 60.0 | 59.8 | .551 | 57.6 | 59.1 | 61.8 | 58.2 | 53.4 | .381 | 59.2 |
| Not able to socialize as wanted^a^ (%) | 1,303 | 61.3 | 62.3 | .776 | 60.9 | 60.9 | 63.1 | .689 | 63.8 | 62.6 | 60.8 | 54.6 | 60.9 | .275 | 61.4 |
| Questing right choice in life^a^ (%) | 795 | 36.5 | 46.2 | .007 | 38.5 | 37.5 | 35.7 | .646 | 47.7 | 36.7 | 37.0 | 32.1 | 34.2 | .006 | 37.4 |
| **CBS Score** (median) | 2,123 | 24.0 | 25.0 | .078^2^ | 24.0 | 24.0 | 24.0 | .925^3^ | 27.0 | 24.0 | 23.0 | 23.0 | 24.0 | <.001^3^ | 24.0 |
| **Subscale Material Burden** (median) | 2,123 | 8.0 | 9.0 | .003^2^ | 8.0 | 8.0 | 8.0 | .256^3^ | 11.0 | 8.0 | 7.0 | 7.0 | 8.0 | <.001^3^ | 8.0 |
| **Subscale Psychosocial Burden** (median) | 2,123 | 15.0 | 15.0 | .699^2^ | 15.0 | 15.0 | 16.0 | .287^3^ | 16.0 | 15.0 | 15.0 | 14.0 | 16.0 | .104^3^ | 15.0 |

^1^ Pearson Chi^2^-Test unless otherwise noted; ^2^ Mann-Whitney-U test; ^3^ Kruskal-Wallis test; ^a^ Includes the response categories: ‘agree somewhat, ‘agree mostly’ and ‘agree completely’; Immigration status: NIH: No immigration history, ID: Immigrants and their (direct) descendants; Educational attainment (CASMIN): B: Basic, M: Medium, H: Higher

Supplementary Table 4_1 Resources during the COVID-19 pandemic differentiated by immigration status, educational attainment (CASMIN) and monthly net household income

| **Characteristics (N=2,123)** | **N** | **Immigration status** | | | **Educational attainment (CASMIN)** | | | | **Monthly net household income** | | | | | | **Total** |
| --- | --- | --- | --- | --- | --- | --- | --- | --- | --- | --- | --- | --- | --- | --- | --- |
|  |  | **NIH** | **ID** | **p^1^** | **B** | **M** | **H** | **p^1^** | **up to under 1,000 euros** | **1,000 to 2,999 euros** | **3,000 to 2,999 euros** | **5,000 euros and more** | **No ans-wer** | **p^1^** |  |
| **CRCS** |  |  |  |  |  |  |  |  |  |  |  |  |  |  |  |
| Continue living normally^a^ (%) | 1,760 | 83.2 | 79.9 | .184 | 82.6 | 83.1 | 82.9 | .584 | 73.3 | 83.0 | 86.8 | 85.2 | 80.1 | <.001 | 82.9 |
| Did things always wanted to do^a^ (%) | 775 | 36.1 | 40.2 | .172 | 33.1 | 35.1 | 44.7 | .002 | 23.0 | 35.1 | 41.3 | 53.6 | 27.3 | <.001 | 36.5 |
| Focused on hobbies^a^ (%) | 795 | 37.4 | 37.7 | .981 | 37.3 | 37.4 | 37.8 | .993 | 34.6 | 37.7 | 36.1 | 45.4 | 35.4 | .230 | 37.4 |
| More in touch with family^a^ (%) | 682 | 31.8 | 35.7 | .347 | 31.7 | 31.4 | 34.4 | .430 | 22.2 | 31.9 | 36.8 | 35.7 | 27.3 | <.001 | 32.1 |
| More in touch with friends^a^ (%) | 512 | 24.0 | 25.1 | .400 | 22.4 | 24.0 | 27.0 | .186 | 19.3 | 24.1 | 25.7 | 28.6 | 20.5 | .494 | 24.1 |
| Better care of personal health^a^ (%) | 654 | 30.6 | 33.2 | .613 | 25.7 | 32.0 | 35.7 | .004 | 22.2 | 29.7 | 33.5 | 44.4 | 24.2 | <.001 | 30.8 |
| Personal activities online^a^ (%) | 610 | 28.5 | 31.2 | .460 | 21.1 | 29.5 | 38.4 | <.001 | 19.8 | 27.3 | 32.5 | 40.3 | 23.0 | <.001 | 28.7 |
| More time to spend with family^a^ (%) | 763 | 35.4 | 40.7 | .116 | 34.0 | 36.3 | 38.0 | .570 | 21.8 | 33.5 | 42.2 | 48.5 | 34.2 | <.001 | 35.9 |
| Develop new routines^a^ (%) | 606 | 28.8 | 25.6 | .256 | 24.5 | 28.3 | 35.1 | <.001 | 19.3 | 26.3 | 32.6 | 44.4 | 21.7 | <.001 | 28.5 |
| More time in nature^a^ (%) | 947 | 44.8 | 42.7 | .841 | 39.4 | 45.8 | 49.7 | .005 | 30.0 | 41.8 | 50.2 | 56.6 | 48.4 | <.001 | 44.6 |
| Time to pause^a^ (%) | 884 | 41.8 | 39.7 | .344 | 37.7 | 42.4 | 45.6 | .066 | 32.9 | 40.1 | 46.7 | 48.5 | 37.3 | .001 | 41.6 |
| Time to relax^a^ (%) | 907 | 42.4 | 45.7 | .574 | 41.7 | 45.4 | 38.2 | .081 | 38.7 | 43.4 | 45.3 | 45.9 | 31.7 | .109 | 42.7 |
| Help from others^a^ (%) | 451 | 21.1 | 22.6 | .322 | 18.9 | 20.4 | 26.5 | .037 | 19.3 | 19.4 | 21.9 | 32.1 | 19.3 | .015 | 21.2 |
| Supported others^a^ (%) | 528 | 25.1 | 23.1 | .051 | 19.1 | 26.9 | 29.0 | <.001 | 13.2 | 23.5 | 27.6 | 36.2 | 26.7 | <.001 | 24.9 |
| **CRCS score** (median) | 2,123 | 35.0 | 35.0 | .461^2^ | 35.0 | 35.0 | 36.0 | .002^3^ | 33.0 | 35.0 | 36.0 | 39.0 | 33.0 | <.001^3^ | 35.0 |
| **CRCS Subscale Self-focused coping strategies** (median) | 2,123 | 21.0 | 21.0 | .756^2^ | 21.0 | 22.0 | 22.0 | .018^3^ | 20.0 | 21.0 | 22.0 | 24.0 | 21.0 | <.001^3^ | 21.0 |
| **CRCS Subscale Social engagement** (median) | 2,123 | 13.0 | 14.0 | .164^2^ | 13.0 | 13.0 | 14.0 | <.001^3^ | 12.0 | 13.0 | 14.0 | 14.0 | 13.0 | <.001^3^ | 13.0 |
| **General self-efficacy (GSE)** |  |  |  |  |  |  |  |  |  |  |  |  |  |  |  |
| **High GSE**^b^ (%) | 1,044 | 49.1 | 49.7 | .865 | 48.6 | 47.3 | 54.4 | .041 | 32.9 | 48.2 | 56.3 | 54.1 | 48.4 | <.001 | 49.2 |
| **GSE** (median) | 2,123 | 29.0 | 29.0 | .747^2^ | 29.0 | 29.0 | 30.0 | .008^3^ | 28.0 | 29.0 | 30.0 | 30.0 | 29.0 | <.001^3^ | 29.0 |
| **GSE** (mean (Std. dev.) | 2,123 | 28.5 (5.7) | 28.5 (6.0) |  | 28.2 (6.2) | 28.3 (5.6) | 29.3 (5.2) |  | 26.2 (6.2) | 28.3 (5.7) | 29.4 (5.3) | 29.7 (5.2) | 27.9 (5.7) |  | 28.5 (5.7) |

^1^ Pearson Chi^2^-Test unless otherwise noted, ^2^ Mann-Whitney-U test, ^3^ Kruskal-Wallis test, ^a^ Includes the response categories: ‘agree completely’, ‘agree mostly’ and ‘somewhat agree’, ^b^ High GSE >= 29 [90]; CRCS: COVID-19 Pandemic related Resources and Coping Scale; Immigration status: NIH: No immigration history, ID: Immigrants and their (direct) descendants; Educational attainment (CASMIN): B: Basic, M: Medium, H: Higher

Supplementary Table 5_1 Self-rated health and mental well-being stratified by immigration status, educational attainment (CASMIN) and monthly net household income

| **Characteristics (N=2,123)** | **N** | **Immigration status** | | | **Educational attainment (CASMIN)** | | | | | **Monthly net household income** | | | | | | **Total** |
| --- | --- | --- | --- | --- | --- | --- | --- | --- | --- | --- | --- | --- | --- | --- | --- | --- |
|  |  | **NIH** | **ID** | **p^1^** | **B** | **M** | **H** | **p^1^** | **up to under 1,000 euros** | | **1,000 to 2,999 euros** | **3,000 to 4,999 euros** | **5,000 euros and more** | **No ans-wer** | **p^1^** |  |
| **Self-rated health** |  |  |  | .571 |  |  |  | <.001 |  | |  |  |  |  | <.001 |  |
| Rather poor^a^ | 1,118 | 52.9 | 50.8 |  | 67.5 | 50.3 | 35.7 |  | 73.7 | | 60.1 | 38.9 | 30.1 | 54.0 |  | 52.7 |
| Rather good^b^ | 1,005 | 47.1 | 49.2 |  | 32.5 | 49.7 | 64.3 |  | 26.3 | | 39.9 | 61.1 | 69.9 | 46.0 |  | 47.3 |
| **Chronic disease** | 1,070 | 51.5 | 39.7 | .002 | 62.0 | 50.6 | 32.6 | <.001 | 71.2 | | 56.5 | 40.6 | 26.0 | 47.8 | <.001 | 50.4 |
| **Mental well-being (WHO-5)** |  |  |  | .477 |  |  |  | <.001 |  | |  |  |  |  | <.001 |  |
| Low mental well-being^c^ | 804 | 37.6 | 40.2 |  | 42.3 | 40.3 | 25.8 |  | 57.2 | | 40.2 | 33.0 | 20.4 | 33.5 |  | 37.9 |
| High mental well-being^d^ | 1,319 | 62.4 | 59.8 |  | 57.7 | 59.7 | 74.2 |  | 42.8 | | 59.8 | 67.0 | 79.6 | 66.5 |  | 62.1 |
| Total score (median) | 2,123 | 15.0 | 14.0 | .864^2^ | 14.0 | 14.0 | 16.0 | <.001^3^ | 11.0 | | 14.0 | 16.0 | 17.0 | 15.0 | <.001^3^ | 15.0 |
| Total score (mean (std. dev.)) | 2,123 | 13.8 (6.1) | 13.8 (5.9) |  | 13.1 (6.5) | 13.6 (6.1) | 15.2 (5.6) |  | 10.9 (6.3) | | 13.5 (6.1) | 14.5 (5.9) | 16.1 (5.2) | 14.2 (6.1) |  | 13.8 (6.1) |

^1^ Pearson Chi^2^-Test unless otherwise noted, ^2^ Mann-Whitney-U test, ^3^ Kruskal-Wallis test, ^a^ Includes the response categories: ‘satisfactory’, ‘poor’ and ‘very poor’, ^b^ Includes the response categories: ‘good’ and ‘very good’, ^c^ Total score below 13 [93], ^d^ Total score of 13 or higher [93]; Immigration status: NIH: No immigration history, ID: Immigrants and their (direct) descendants; Educational attainment (CASMIN): B: Basic, M: Medium, H: Higher
